# Supplementary material for: Metabolic coupling between soil aerobic methanotrophs and denitrifiers in rice paddy fields
Source: Nat Commun. 2024 Apr 24;15:3471. doi: 10.1038/s41467-024-47827-y (PMC11043409; doi:10.1038/s41467-024-47827-y)
Supplement: Supplementary file 1 — Supplementary Information [file 41467_2024_47827_MOESM1_ESM.pdf]

Supplementary Information for

Metabolic coupling between soil aerobic methanotrophs and denitrifiers in rice paddy fields

Kang-Hua Chen[1,2], Jiao Feng[1,2*], Paul L. E. Bodelier[3], Ziming Yang[4], Qiaoyun Huang[1,2], Manuel Delgado-Baquerizo[5], Peng Cai[1,2], Wenfeng Tan[2], Yu-Rong Liu[1,2*]

[1]National Key Laboratory of Agricultural Microbiology and College of Resources and Environment, Huazhong Agricultural University, Wuhan, 430070, China

[2]State Environmental Protection Key Laboratory of Soil Health and Green Remediation and Hubei Key Laboratory of Soil Environment and Pollution Remediation, Huazhong Agricultural University, Wuhan, 430070, China

[3]Department of Microbial Ecology, Netherlands Institute of Ecology (NIOO-KNAW), PO Box 50, 6700 AB, Wageningen, The Netherlands

[4]Department of Chemistry, Oakland University, Rochester, MI, 48309, USA

[5]Laboratorio de Biodiversidad y Funcionamiento Ecosistémico, Instituto de Recursos Naturales y Agrobiología de Sevilla (IRNAS), CSIC, Sevilla, 41012, Spain

***Corresponding authors**

Jiao Feng; E-mail: fengjiao@mail.hzau.edu.cn;

Yu-Rong Liu; E-mail: yrliu@mail.hzau.edu.cn

This PDF file includes:

Supplementary Figs. 1 to 20

Supplementary Tables 1 to 7

Supplementary References

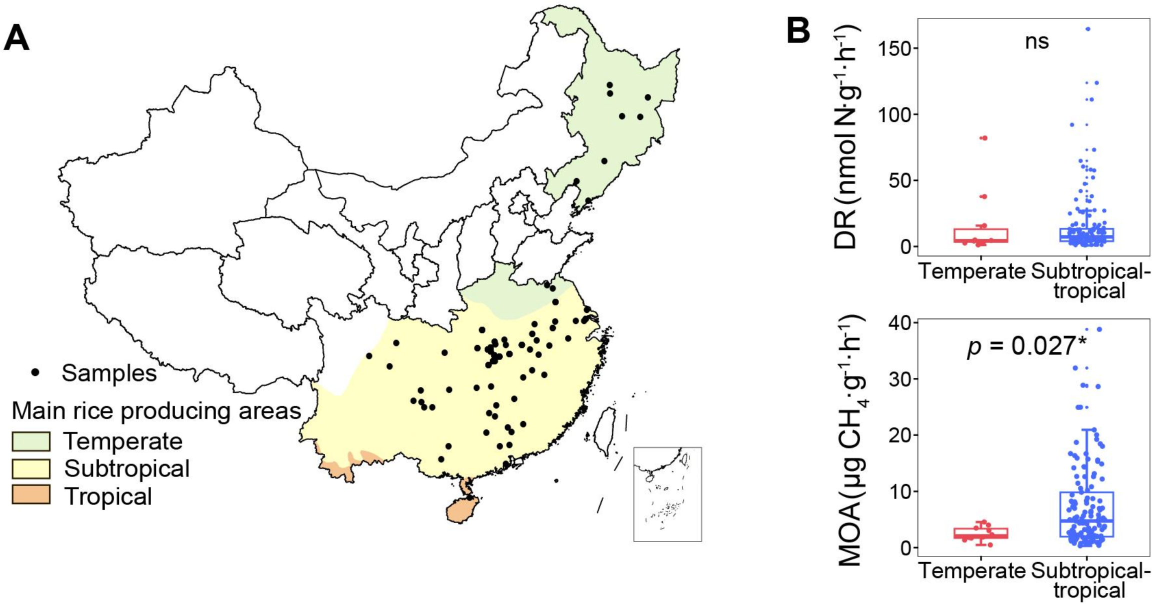

Supplementary Fig. 1 The distribution of sampling sites and the activities of denitrification (DR) and methane oxidation (MOA) across main rice-producing areas of China. **A** A total of 139 representative soil samples were collected, spanning a > 3300 km transect from the northeast to the south of China. The transect encompasses diverse climatic zones, including temperate, subtropical and tropical regions; **B** The differences of DR and MOA between temperate and subtropical-tropical regions. The box plots display the interquartile range, comprising the first quartile, median, and third quartile, while the whiskers extend from the minimum to the maximum values. * indicates statistically significant differences based on the two-sided T-Test of $p < 0.05$. Source data are provided as a Source Data file.

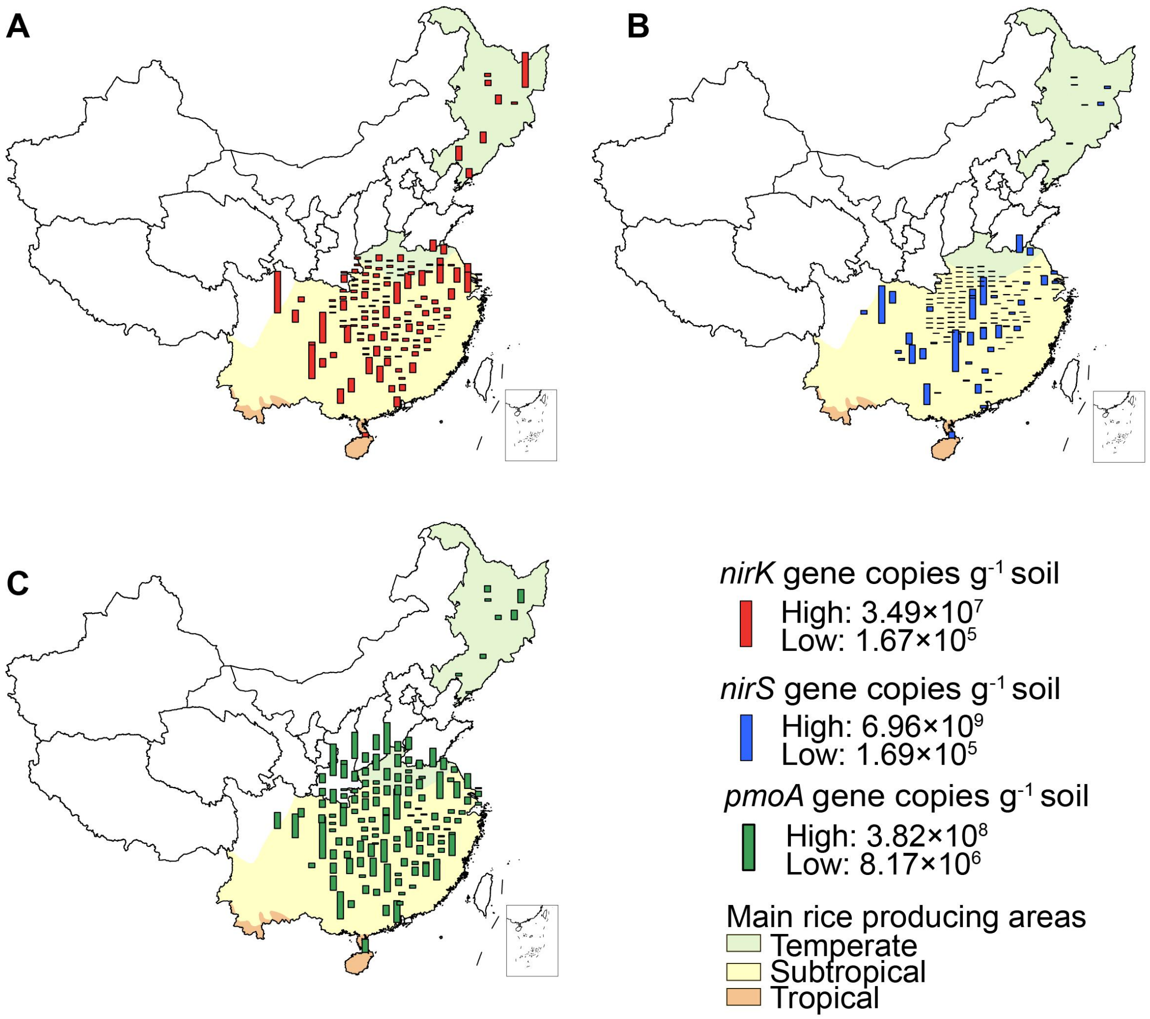

Supplementary Fig. 2 The abundance of genes associated with denitrification (*nirK* and *nirS*) and methane oxidation (*pmoA*) across climate zones of main rice-producing areas of China. A-C The distribution patterns of *nirK*, *nirS* and *pmoA* genes.

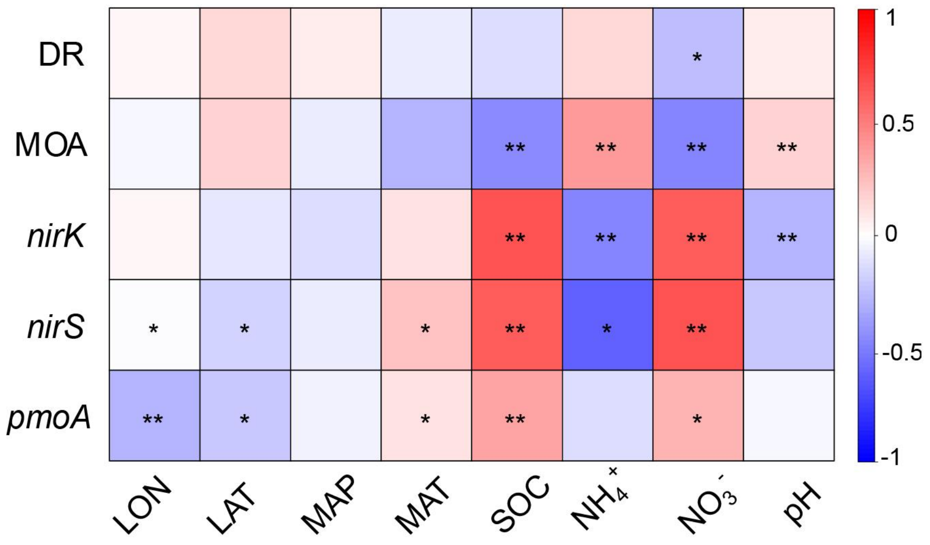

Supplementary Fig. 3 Pearson correlations of activities and genes associated with methane (CH_4) oxidation and denitrification with environmental factors. DR, denitrification rate; MOA, CH_4-oxidizing activity; LON, longitude; LAT, latitude; MAP, mean annual precipitation; MAT, mean annual temperature; SOC, soil organic carbon. * indicated $p < 0.05$ and ** indicated $p < 0.01$. Exact *p*-values and Source data are provided as a Source Data file.

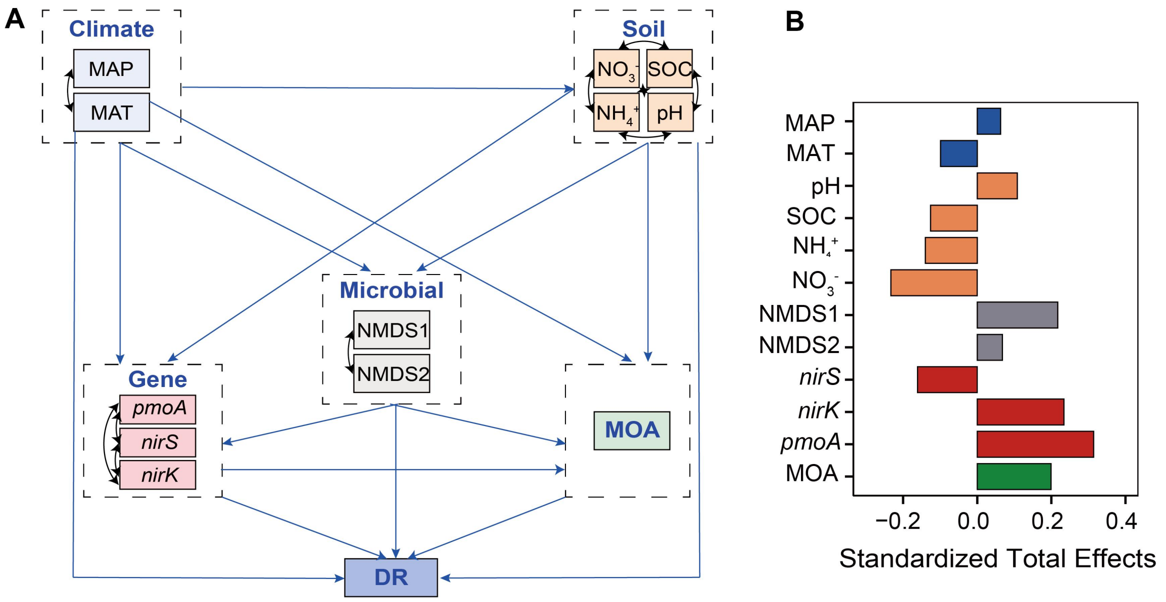

Supplementary Fig. 4 The rationale for the associations of environmental factors and their influences on soil denitrification rate (DR). A A priori generic structural equation model used in this study; **B** The standardized total effects of different environmental variables on DR. The model evaluated the effects of climatic (MAP and MAT), soil properties (SOC, NH_4^+, NO_3^- and soil pH), methane-oxidizing activity (MOA) and microbial attributes (NMDS1, NMDS2 and functional gene abundances) on DR. MAP, mean annual precipitation; MAT, mean annual temperature; SOC, soil organic carbon; NMDS1 and NMDS2 represents the two axes of a nonmetric multidimensional scaling (NMDS) analysis. Source data are provided as a Source Data file.

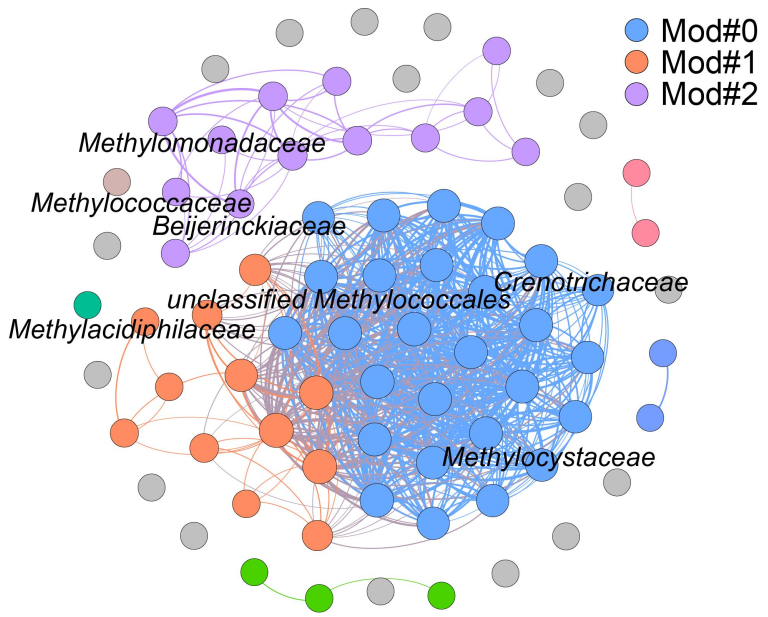

Supplementary Fig. 5 Methanotrophs associated with the denitrification process. Network analyses showing the co-occurrence patterns among methanotrophs and denitrifiers. A total of 71 denitrifying and 7 methanotrophic families co-occurred significantly across the main rice-producing areas of China. Source data are provided as a Source Data file.

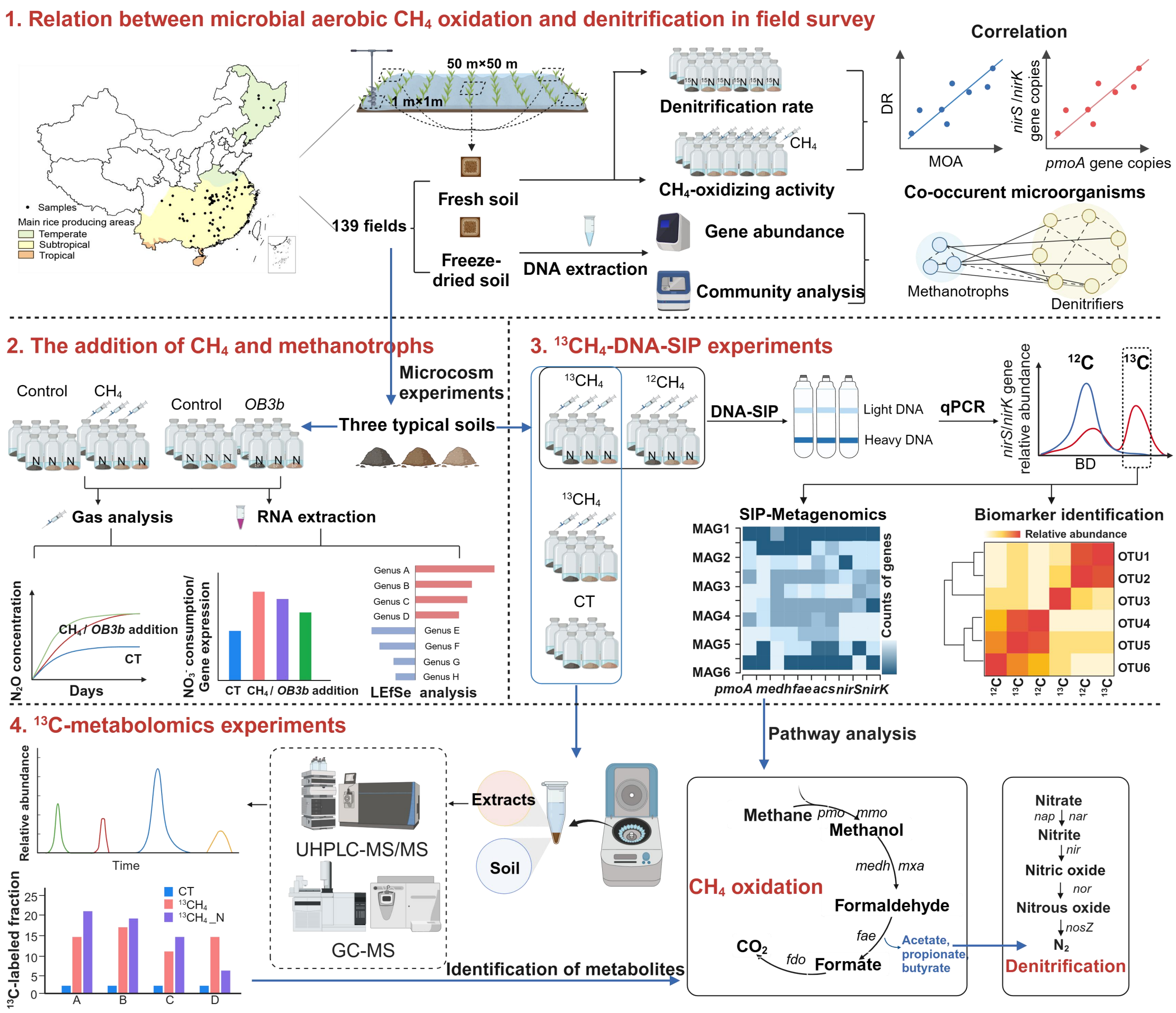

Supplementary Fig. 6 Workflow of the field survey and microcosm experiments in rice paddy fields.

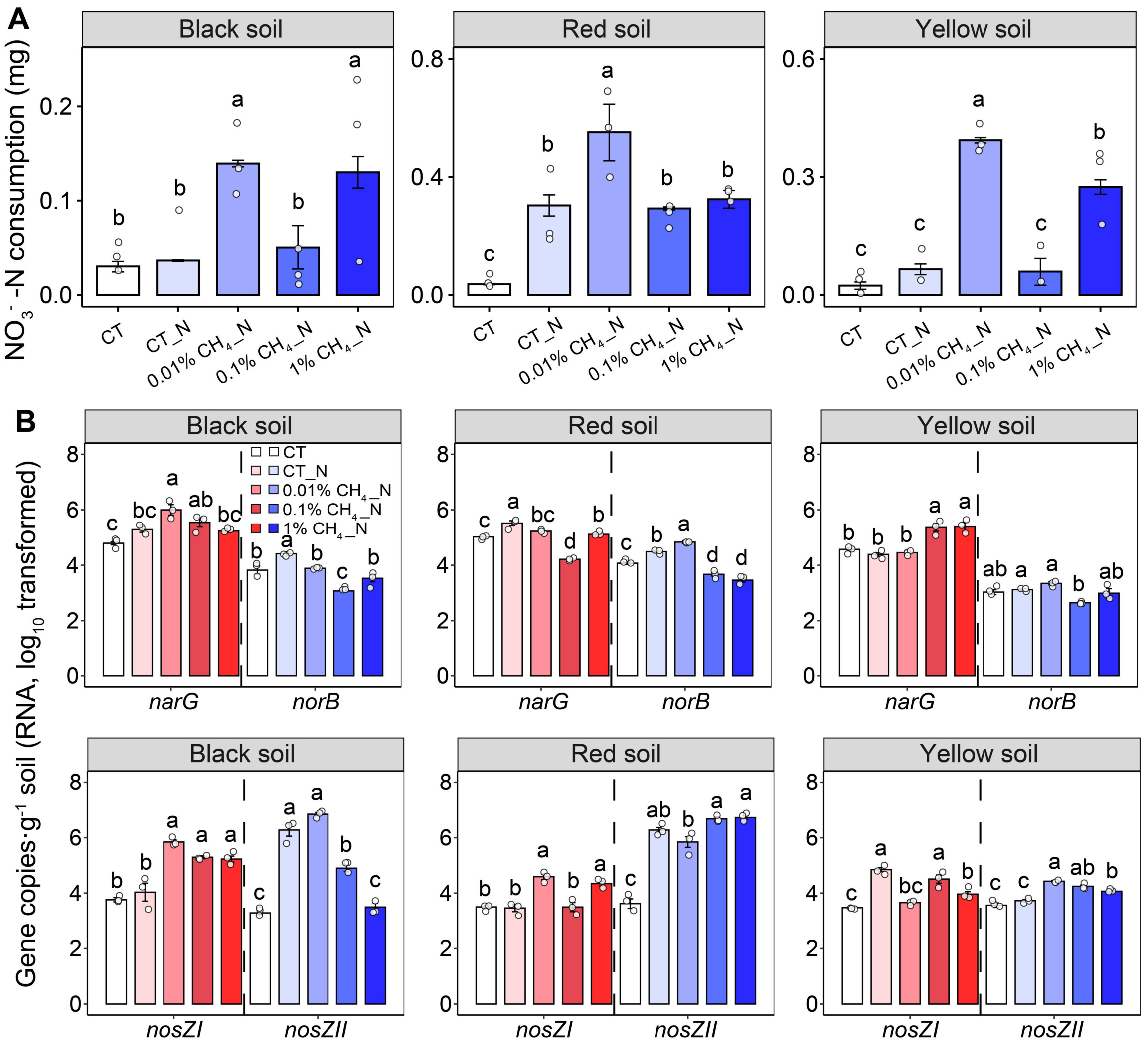

Supplementary Fig. 7 Effects of methane (CH_4) addition on microbial denitrification of three typical paddy soils. **A** Variations in consumption of nitrate (NO_3^--N); and **B** Changes in denitrification gene (*narG*, *norB*, *nosZI* and *nosZII*) expression using RNA reverse transcription. The error bar represents the standard error of triplicate samples, and data are presented as mean values ± standard error. Different lowercase letters indicate significant differences between the soils with different CH_4 concentrations ($p < 0.05$; n = 3; one-way ANOVA followed by two-sided Tukey post hoc test). Exact p-values and Source data are provided as a Source Data file.

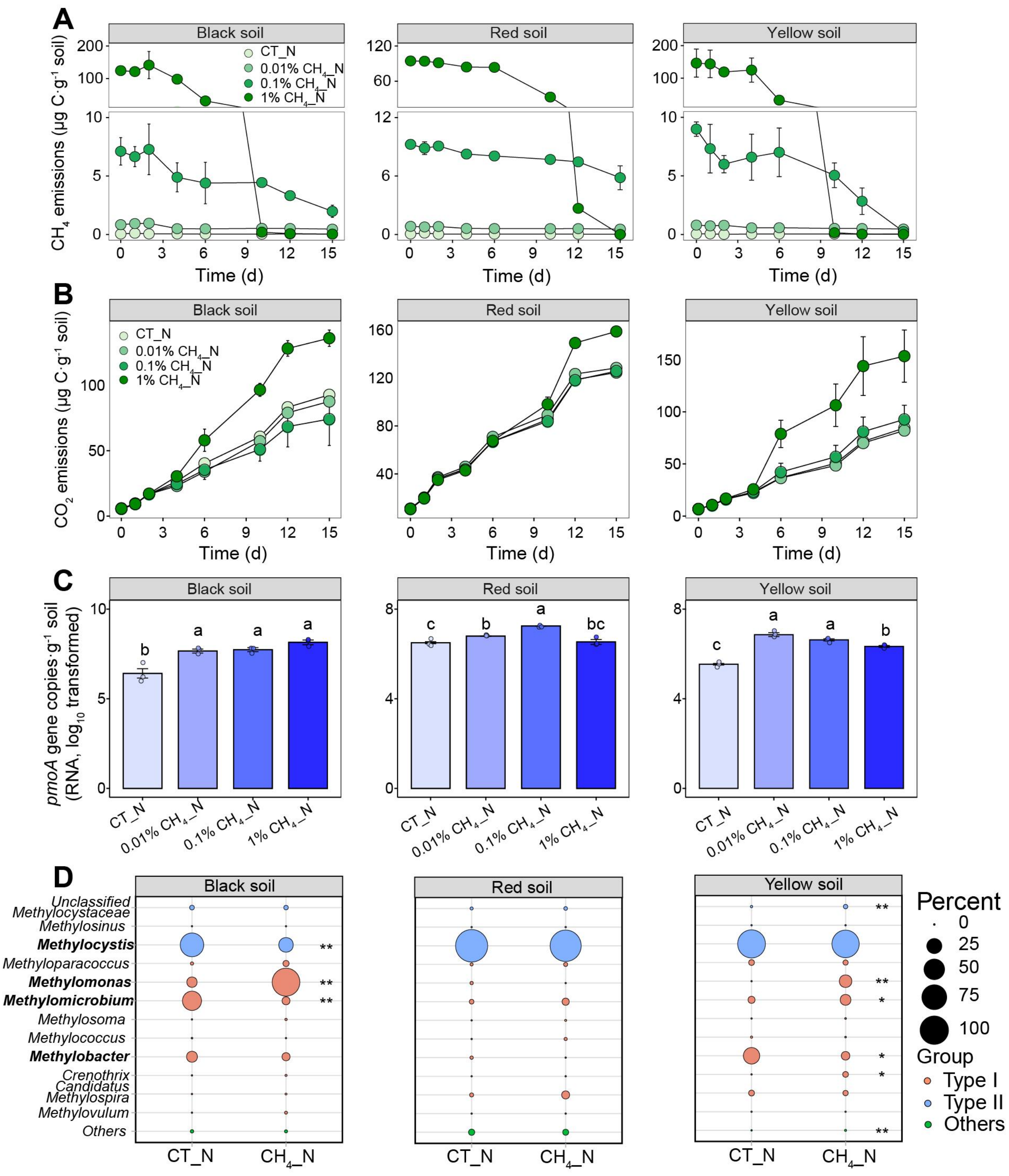

Supplementary Fig. 8 Effects of methane (CH_4) addition on microbial CH_4 oxidation of three typical paddy soils. A-B Variations in CH_4 and carbon dioxide (CO_2) emissions; **C-D** Changes in *pmoA* gene transcription and key genera of methanotrophs using RNA reverse transcription in CH_4 addition experiments. The error bar in (A-C) represents the standard error of triplicate samples, and data are presented as mean values ± standard error. Different lowercase letters in (C) indicate significant differences between the soils with different CH_4 concentrations ($p < 0.05$; n = 3; one-way ANOVA followed by two-sided Tukey post hoc test). * indicates statistically significant levels of $p < 0.05$ and ** indicates $p < 0.01$ based on two-sided T-Test in (D). Exact p-values and Source data are provided as a Source Data file.

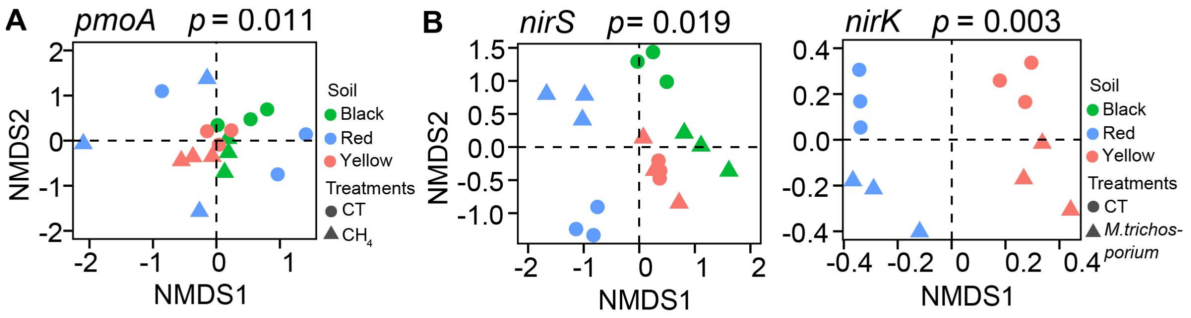

Supplementary Fig. 9 Effects of addition of methane (CH_4) and methanotrophs on the composition of functional genes involved in CH_4 oxidation and denitrification. A The effects of CH_4 addition on bacterial beta-diversity in CH_4 oxidizing gene (*pmoA*); **B** Methanotrophs addition affects bacterial beta-diversity of denitrification genes (*nirS* and *nirK*) by permutational multivariate analysis of variance (PERMANOVA). Source data are provided as a Source Data file.

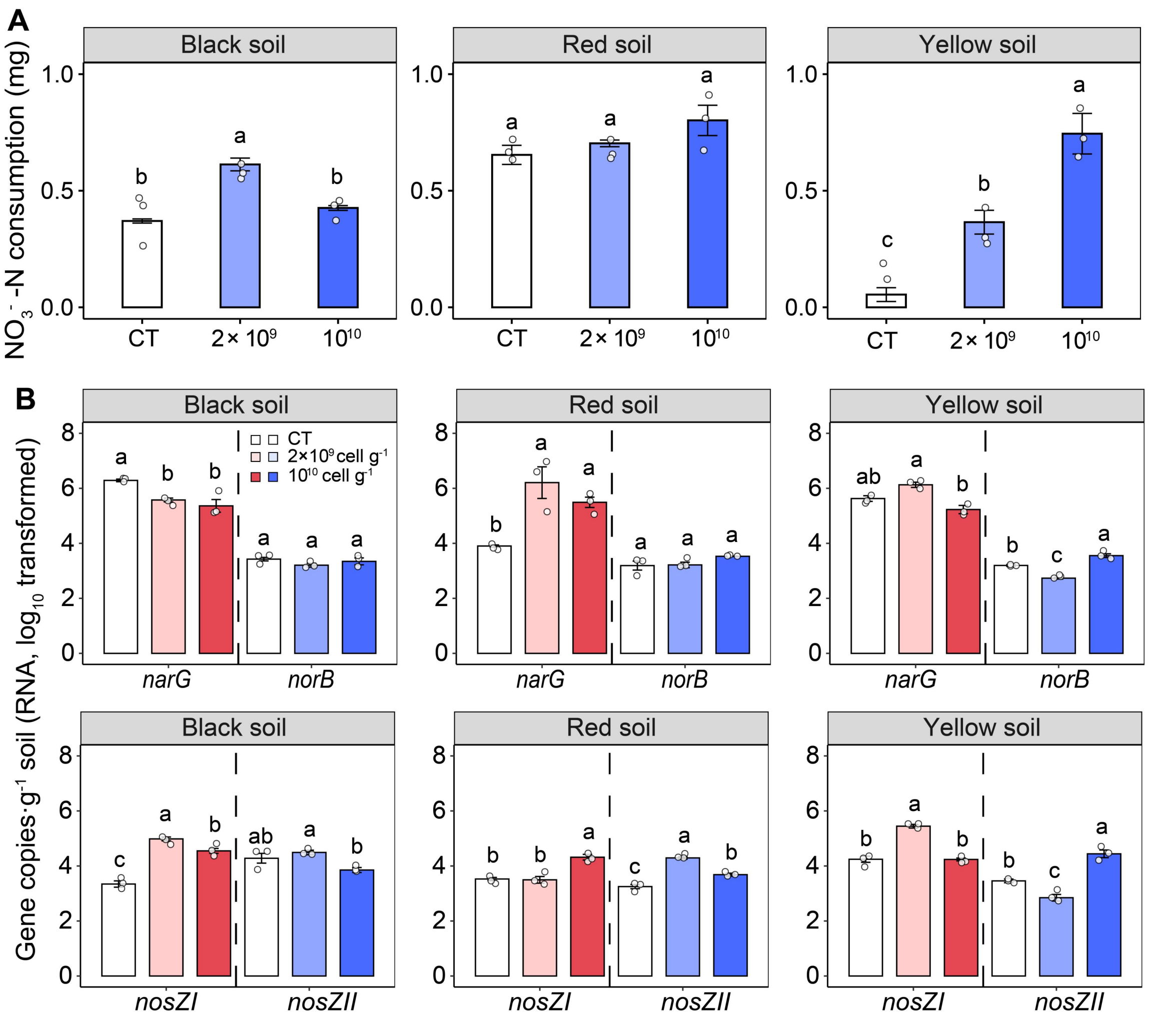

Supplementary Fig. 10 Effects of addition of aerobic methanotrophs on soil denitrification. **A** Variations in consumption of nitrate (NO_3^--N); **B** Changes in the transcript of denitrification genes (*narG*, *norB*, *nosZI* and *nosZII*) based on RNA reverse transcription. The error bar represents the standard error of triplicate samples, and data are presented as mean values ± standard error. Different lowercase letters indicate significant differences between soils with different amounts of aerobic methanotrophs added ($p < 0.05$; n = 3; one-way ANOVA followed by two-sided Tukey post hoc test). Exact p-values and Source data are provided as a Source Data file.

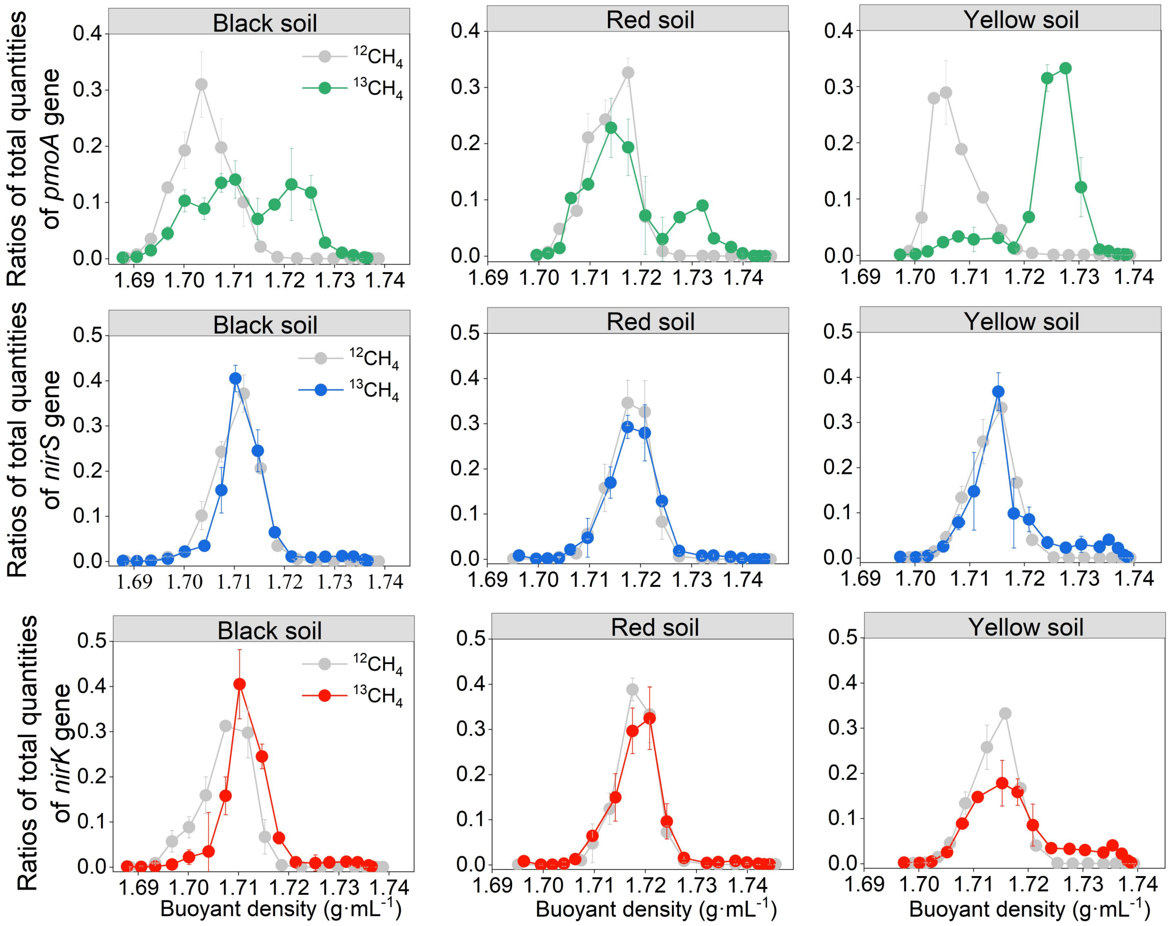

Supplementary Fig. 11 Quantitative distribution of genes involved in methane (CH_4) oxidation (*pmoA*) and denitrification (*nirS* and *nirK*) in eighteen fractions from each CsCl gradient in the three soils. Quantitative distribution of the *pmoA*, *nirS* and *nirK* genes in eighteen fractions from each CsCl gradient, covering a density range from 1.69 to 1.75 g mL^{-1} of the DNA fractions from three typical soils incubated with $^{12}CH_4$ and $^{13}CH_4$ for 15 days. The error bar represents the standard error of triplicate samples, and data are presented as mean values ± standard error. Source data are provided as a Source Data file.

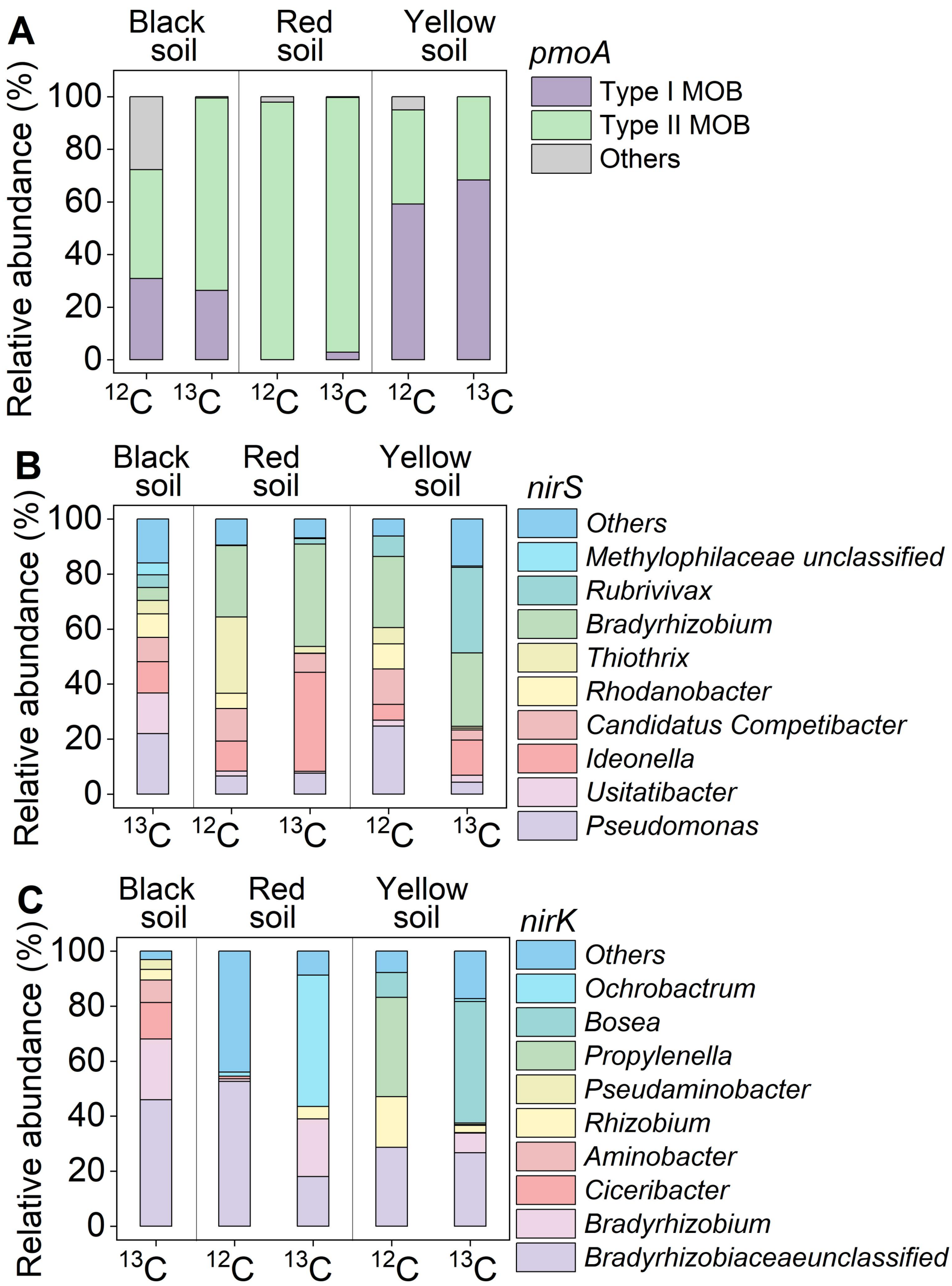

Supplementary Fig. 12 Community composition of methanotrophs and denitrifiers in the ^{12}C-methane (CH_4) and ^{13}C-CH_4 treatments in three typical soils. A Dominant types of methanotrophs in the ^{12}C-CH_4 and ^{13}C-CH_4 treatments in three typical soils; **B-C** Relative abundance of the top ten genera of *nirS*-denitrifiers and *nirK*-denitrifiers in the ^{12}C-CH_4 and ^{13}C-CH_4 treatments. Source data are provided as a Source Data file.

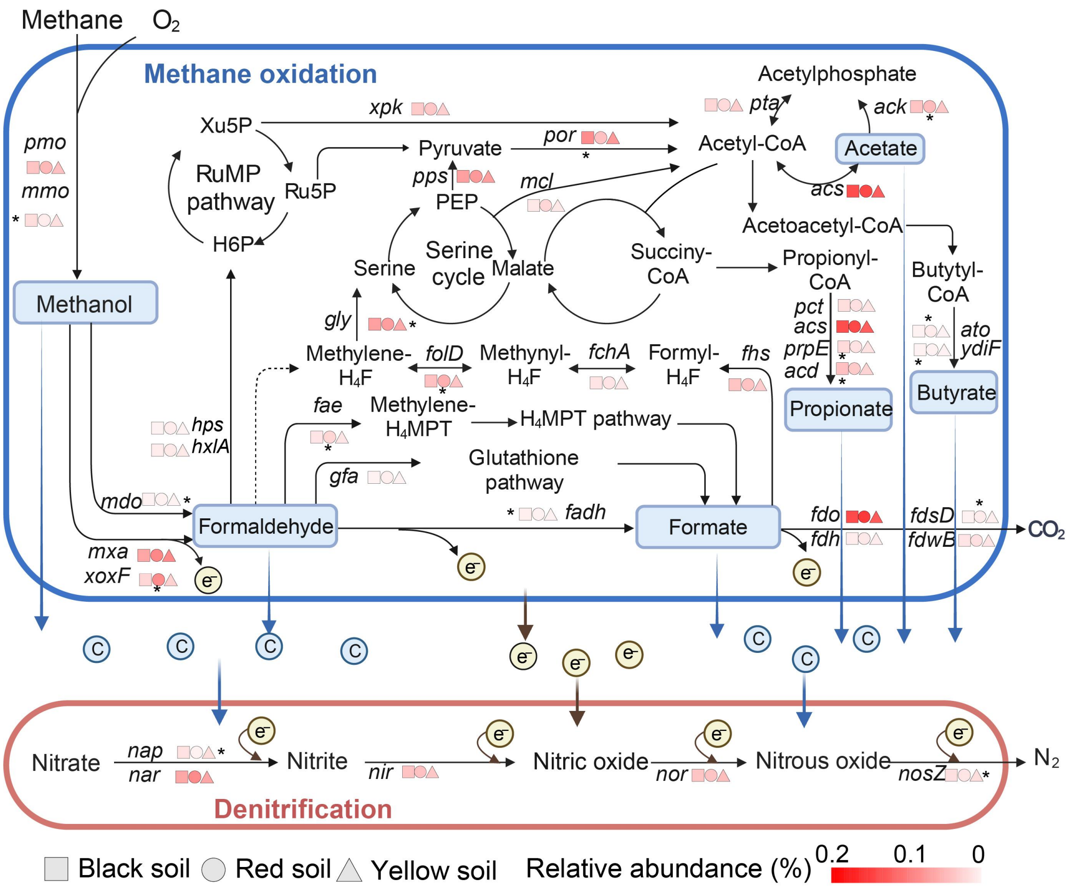

Supplementary Fig. 13 The proposed metagenomic pathways of the coupling between aerobic methane (CH_4) oxidation and denitrification at the community level in the paddy soils. The color gradient represents the relative abundances of major genes of metagenomic analysis in the heavy DNA from $^{13}CH_4$ incubation. * indicates statistically significant differences in the three selected soils at levels of $p < 0.05$ based on one-way ANOVA followed by two-sided Tukey post hoc test. The square, circle, and triangle represent genes in black soil, red soil, and yellow soil, respectively. The definitions of abbreviations are listed in Supplementary Table 6. Source data are provided as a Source Data file.

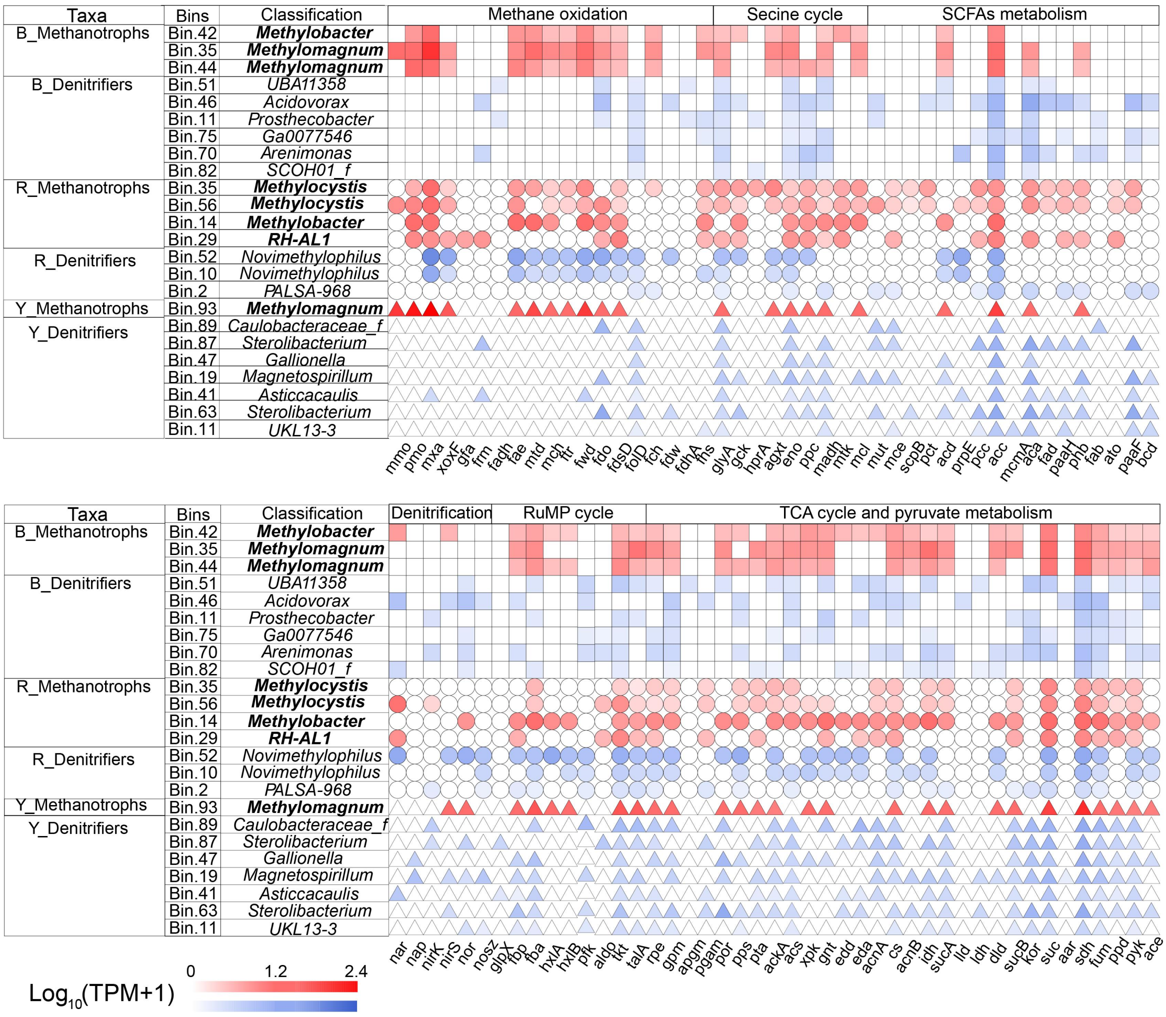

Supplementary Fig. 14 The counts of the major genes for methane (CH_4) oxidation, denitrification and related metabolic pathways in three typical soils. The color gradient represents the logarithm of transcripts per million (TPM) of major genes in the corresponding metagenome-assembled genomes (MAGs) classified to methanotrophs and denitrifiers, respectively, in the heavy DNA from $^{13}CH_4$ incubation. The counts of genes were log (n+1) transformed. The square, circle, and triangle represent MAGs recovered from black soil, red soil, and yellow soil, respectively. The red and blue symbols represent MAGs belonged to methanotrophs and denitrifiers, respectively. The definitions of abbreviations are listed in Supplementary Table 7. Source data are provided as a Source Data file.

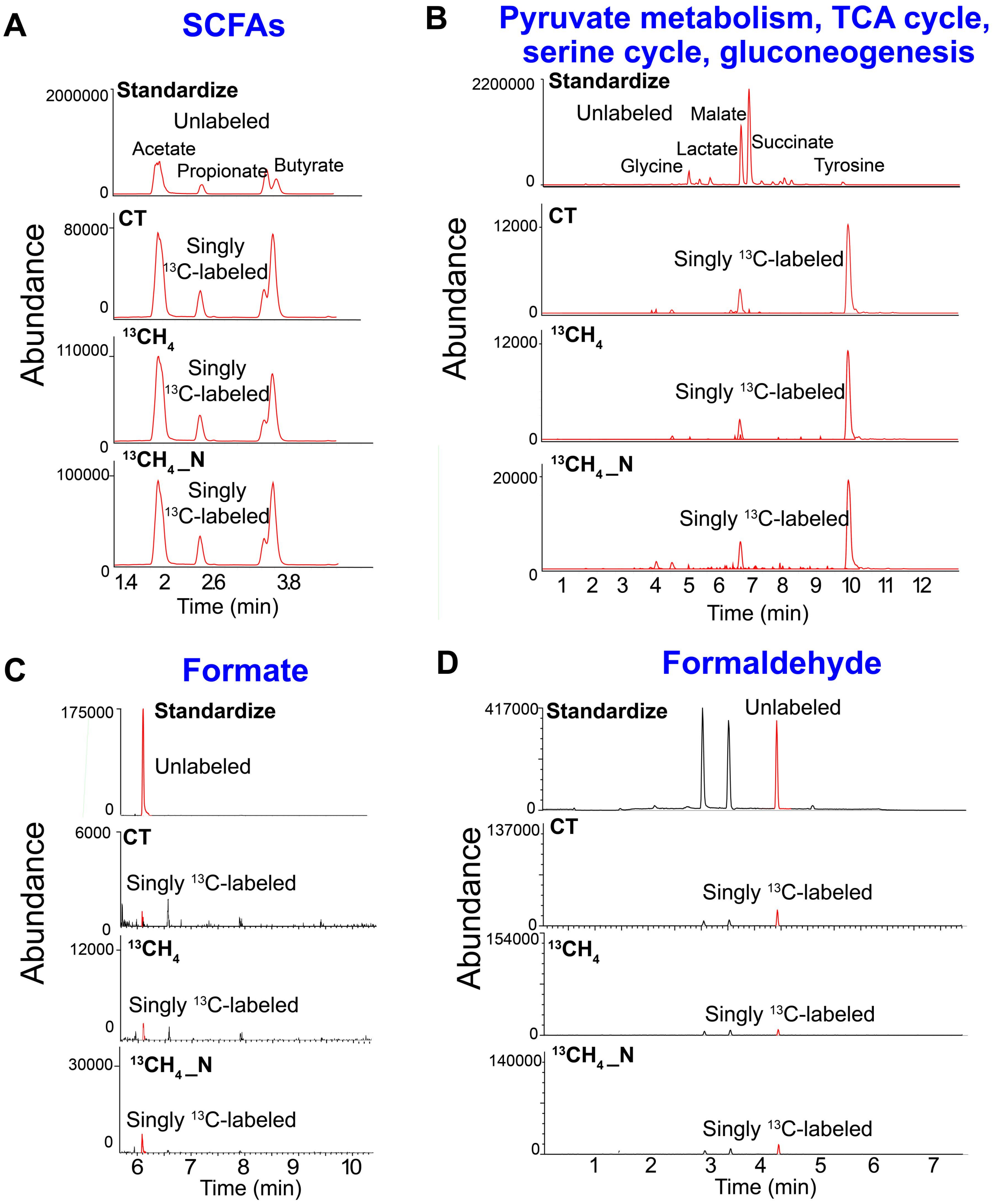

123 **Supplementary Fig. 15 Typical LC–MS/MS and GC-MS chromatograms of targeted**
124 **metabolites originating from methane (CH_4) oxidation in three typical soils**. **A** Short
125 chain fatty acids (SCFAs); **B** The intermediates involved in pyruvate metabolism, TCA cycle,
126 serine cycle and gluconeogenesis; **C** Formate; **D** Formaldehyde.

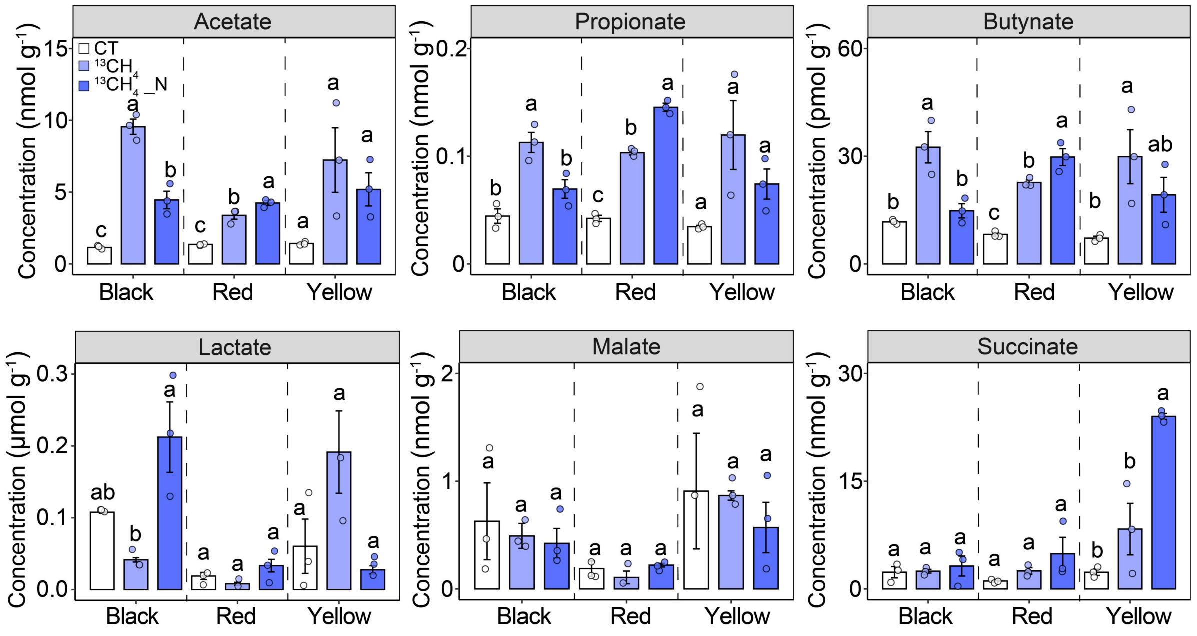

Supplementary Fig. 16 The concentration of metabolites originating from methane (CH_4) oxidation in three typical soils. The error bar in (B) represents the standard error of triplicate samples, and data are presented as mean values ± standard error (n = 3; one-way ANOVA followed by two-sided Tukey post hoc test). Different lowercase letters in (B) indicate significant differences between treatments (*p* < 0.05). Exact *p*-values and Source data are provided as a Source Data file.

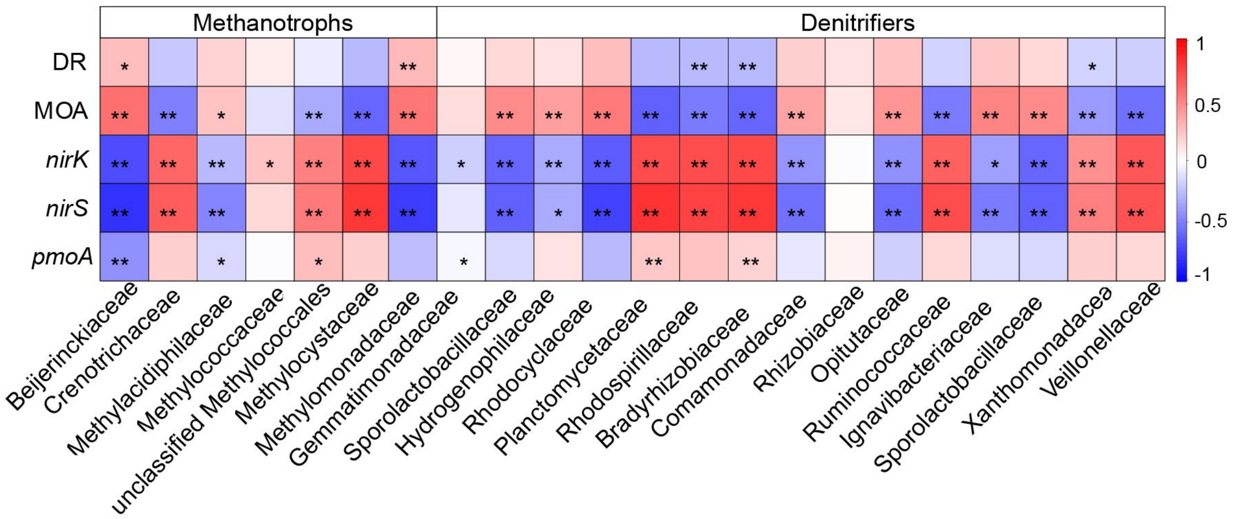

Supplementary Fig. 17 Pearson correlations of activities and genes with key taxa related to methane (CH_4) oxidation and denitrification. DR, denitrification rate; MOA, CH_4 oxidizing activity. Significant correlations are indicated by *. * indicated $p < 0.05$ and ** indicated $p < 0.01$. Source data are provided as a Source Data file.

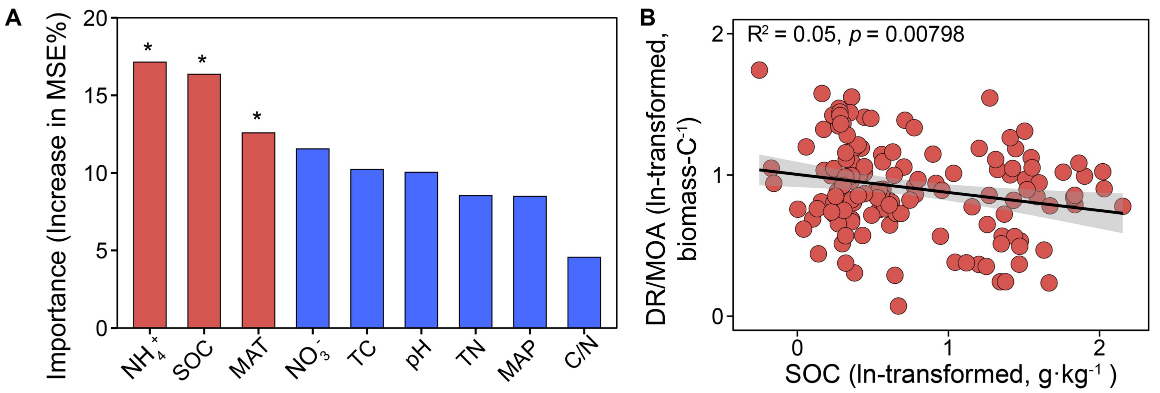

Supplementary Fig. 18 Factors predicting the relationships between methane oxidation and denitrification. A Random forest analysis identifying significant predictors on the relative denitrification to methane oxidation activities (DR/MBC)/ (MOA/MBC) (p < 0.05); **B** The relationships between (DR/MBC)/ (MOA/MBC) and SOC. SOC, soil organic carbon; MAT, mean annual temperature; MAP, mean annual precipitation. Exact p-values in (A) and Source data are provided as a Source Data file.

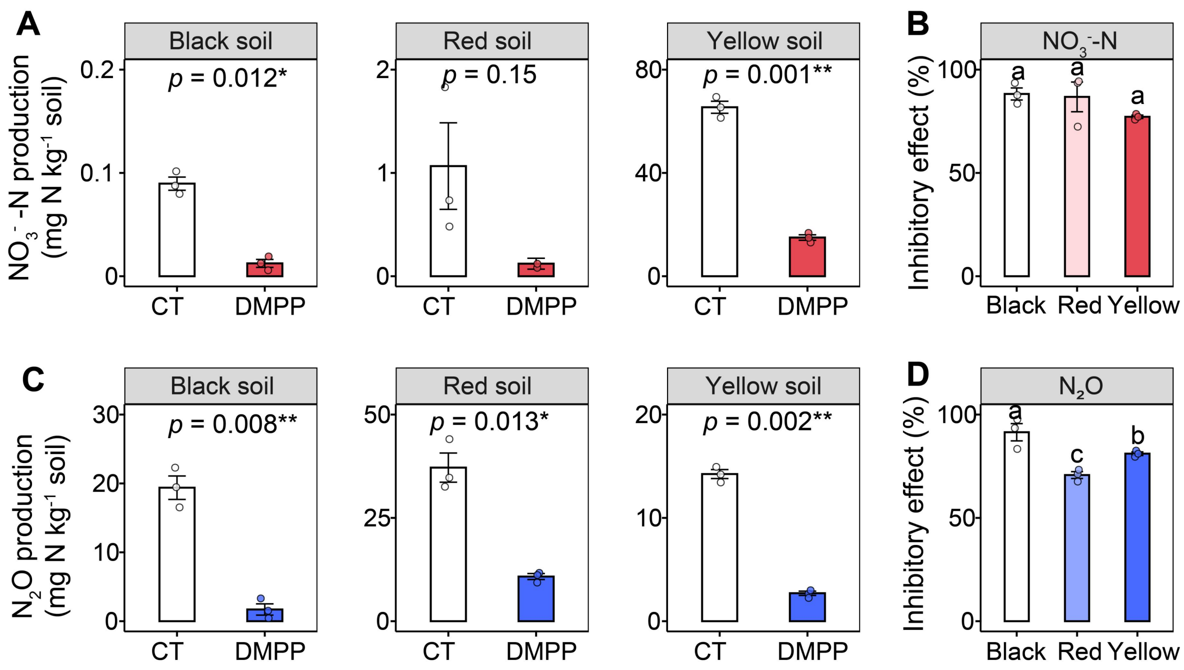

Supplementary Fig. 19 The inhibition effects of 3,4-dimethylpyrazole phosphate (DMPP) on nitrification in the three soils. **A** Variations in production of nitrate (NO_3^-); **B** The inhibitory effects of DMPP on NO_3^- production; **C** Changes in nitrous oxide (N_2O) emissions; **D** The inhibitory effects of DMPP on N_2O emissions. The error bar represents the standard error of triplicate samples, and data are presented as mean values ± standard error. * indicates statistically significant levels of $p < 0.05$ and ** indicates $p < 0.01$ based on two-sided T-Test in (A) and (C). Different lowercase letters in (B) and (D) indicate significant differences between treatments ($p < 0.05$; one-way ANOVA followed by two-sided Tukey post hoc test). Exact p-values and Source data are provided as a Source Data file.

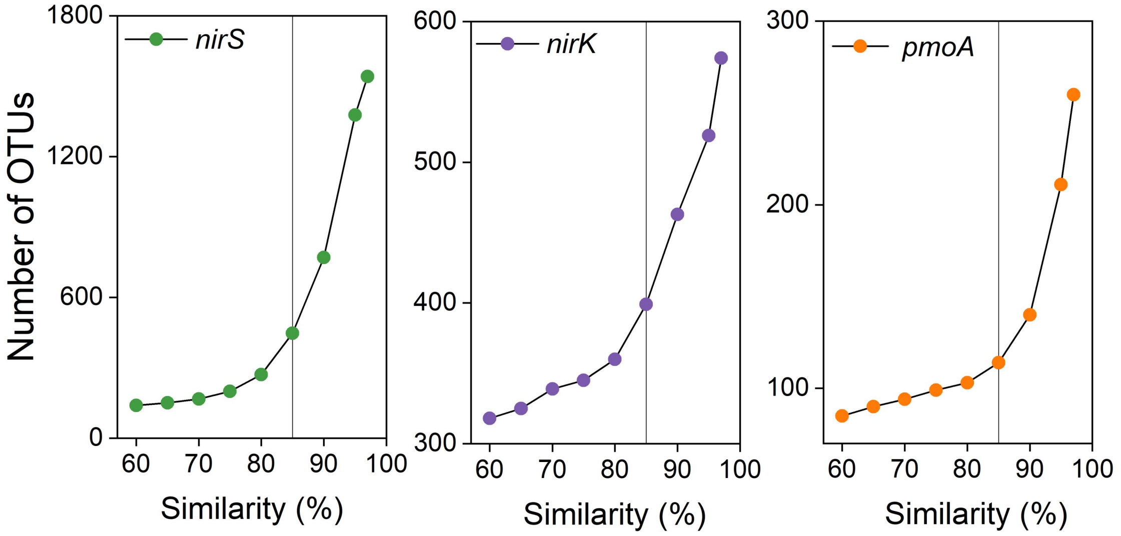

Supplementary Fig. 20 The counts of operational taxonomic units (OTUs) clustering at various thresholds. Source data are provided as a Source Data file.

Supplementary Table 1. Geographical information and physicochemical characteristics of collected natural soil samples.

Sample	Longitude (°)	Latitude (°)	TC (%)	TN (%)	pH	Clay (g kg^{-1})	SOC (g kg^{-1})
Black soil	126.63	47.43	3.76	0.28	6.43	334	37.53
Red soil	112.83	28.56	2.26	0.21	4.69	227	23.08
Yellow soil	113.36	23.16	1.31	0.12	5.68	192	14.10

Supplementary Table 2. Properties of recovered metagenome assembled genomes (MAGs) affiliated with methanotrophs and denitrifiers with completeness >50% and contamination <10%.

Bin Id	Contigs	Genome size (bp)	N50	Completeness (%)	Contamination (%)	16S rRNA	23S rRNA	5S rRNA	tRNA	Bin quality	Abundance (%)
**B_Bin42**	298	5242199	27616	98.94	1.63	0	0	1	40	MQ	1.36
**B_Bin35**	145	4715769	73841	98.21	2.13	0	0	0	47	MQ	2.25
**B_Bin44**	708	5957189	12457	95.36	7.94	0	0	0	42	MQ	0.99
**B_Bin46**	200	5283043	60611	94.70	4.87	0	0	0	46	MQ	0.57
**B_Bin51**	257	4855676	31935	97.30	5.07	0	0	1	48	MQ	0.89
**B_Bin11**	1087	6039704	7597	91.16	1.02	0	0	0	40	MQ	0.43
**B_Bin75**	835	5249229	8093	90.31	3.89	0	1	1	45	MQ	0.30
**B_Bin70**	189	2931300	28219	83.10	0.86	0	0	0	41	MQ	0.64
**B_Bin82**	751	3024827	4681	82.91	1.42	0	0	0	30	MQ	0.28
**R_Bin35**	160	3431118	120546	97.44	2.27	0	0	0	45	MQ	0.69
**R_Bin56**	967	7028512	144445	94.68	8.73	0	0	0	63	MQ	0.54
**R_Bin14**	504	3899330	52019	84.69	2.48	0	0	0	32	MQ	5.15
**R_Bin29**	781	3403938	37244	83.75	3.55	0	0	0	35	MQ	1.29
**R_Bin61**	186	3452242	114161	71.23	3.51	0	0	0	26	MQ	5.31
**R_Bin37**	82	2444772	183383	68.94	2.61	0	0	1	33	MQ	2.03
**R_Bin52**	48	3116807	262841	98.72	0.11	0	0	0	40	MQ	4.71
**R_Bin10**	27	2316567	396376	98.29	0.47	0	0	1	41	MQ	0.99
**R_Bin2**	390	3872137	58474	95.48	0.89	0	0	0	32	MQ	0.64
**R_Bin38**	974	2087582	6531	52.15	6.29	0	0	0	5	MQ	0.91
**Y_Bin93**	304	4721736	29398	87.83	7.29	0	0	0	35	MQ	9.07
**Y_Bin80**	127	3945945	50637	75.31	1.08	0	0	0	38	MQ	3.37
**Y_Bin97**	1031	3227355	3397	69.66	3.11	0	0	0	35	MQ	1.56
**Y_Bin89**	44	5674177	202138	99.32	3.42	0	0	1	44	MQ	1.89
**Y_Bin87**	103	3485676	53718	99.18	1.48	0	0	1	42	MQ	1.26
**Y_Bin47**	49	2998556	152796	98.41	2.30	0	0	0	45	MQ	0.92
**Y_Bin19**	336	4254566	23528	96.40	1.55	0	0	0	52	MQ	1.21
**Y_Bin41**	425	4084344	16372	95.73	4.50	0	0	0	43	MQ	0.45
**Y_Bin63**	117	4337290	114236	94.54	5.29	0	0	0	45	MQ	1.08
**Y_Bin11**	734	2187264	3226	98.81	5.02	0	0	1	35	MQ	0.49
**Y_Bin1**	304	4721736	29398	62.99	4.98	0	0	0	17	MQ	0.53

Supplementary Table 3. The determined various intermediates involved in CH_4 oxidation.

Pathway	Metabolite	Formula	Exact mass
C1	Formaldehyde	CH_2O	30.0106
	Formate	CH_2O_2	46.0055
Short-chain fatty acids	Acetate	C2H4O2	60.0211
	Propionate	$C_3H_6O_2$	74.0368
	Butyrate	$C_4H_8O_2$	88.0524
TCA cycle and pyruvate metabolism	Citrate	$C_6H_8O_7$	192.0270
	Succinate	$C_4H_6O_4$	118.0266
	Malate	$C_4H_6O_5$	134.0215
	Oxalate	$C_2H_2O_4$	89.9953
	Lactate	$C_3H_6O_3$	90.0317
	Oxaloacetate	$C_4H_4O_5$	132.0059
	Fumarate	$C_4H_4O_4$	116.0110
Serine cycle	Pyruvate	$C_3H_4O_3$	88.0160
	Glycine	$C_2H_5NO_2$	75.0320
	Serine	$C_3H_7NO_3$	105.0426
	Phosphoenolpyruvate	$C_3H_5O_6P$	167.9824
	Tyrosine	$C_9H_{11}NO_3$	181.0739
	Hydroxypyruvate	$C_3H_4O_4$	104.0110
	D-Glycerate	$C_3H_6O_4$	106.0266
	Glutamine	$C_5H_{10}N_2O_3$	146.0691
	Glutamate	$C_5H_9NO_4$	147.0532
	Alanine	$C_3H_7NO_2$	89.0477
Gluconeogenesis	Fructose 6-phosphate	$C_6H_{13}O_9P$	260.0297
	Glyceraldehyde 3-phosphate	$C_3H_7O_6P$	169.9980
	Ribulose 5-phosphate	$C_5H_{11}O_8P$	230.0192
	Acetyl phosphate	$C_2H_5O_5P$	139.9875
	Xylulose 5-phosphate	$C_5H_{11}O_8P$	230.0192
	Fructose 1,6-bisphosphate	$C_6H_{14}O_{12}P_2$	339.9960
	2-Phospho-D-glycerate	$C_3H_7O_7P$	185.9929
	Glucose	$C_6H_{12}O_6$	180.0634

Supplementary Table 4. Primer sets and amplification programs used in the quantitative PCR.

Gene	Primer	Sequence (5'-3')	Protocol	Ref
pmoA	A189f	GGNGACTGGGACTTCTGG	95°C/3min, 35 cycles (94°C/45s, 53°C/60s, 72°C/2min),72°C/4min	[1, 2]
	mb661r	CCGGMGCAACGTCYTTACC		
nirK	1F	GGMATGGTKCCSTGGCA	95°C/3min, 40 cycles (95°C/30s,59°C/40s,72°C/30s), 72°C/10min	[3]
	5R	GCCTCGATCAGRTTRTGG		
nirS	cd3aF	GTSAACGTSAAGGARACSGG	95°C/3min, 30 cycles (95°C/45s,57°C/45s, 72°C/45s), 72°C/5min	[4]
	R3cd	GASTTCGGRTGSGTCTTGA		
narG	1960m2F	TAYGTSGGGCAGGARAAACTG	95ºC/3 min, 35 cycles (95ºC/10s, 56ºC/30s, 72ºC/20s), 72°C/10min	[5]
	2050m2R	CGTAGAAGAAGCTGGTGCTGTT		
norB	qnorB*2F*	GGNCAYCARGGNTAYGA	95°C/3min, 40 cycles (94°C/15s, 54°C/30s, 72°C/45s),72°C/10min	[6]
	qnorB5R	ACCCANAGRTGNACNACCCACCA		
nosZI	2F	CGCRACGGCAASAAGGTSMSSGT	95°C/3min, 35 cycles (95°C/45s,57°C/45s, 72°C/45s), 72°C/5min	[7]
	2R	CAKRTGCAKSGCRTGGCAGAA		
nosZII	1F	CTIGGICCIYTKCAYAC	95°C/3min, 40 cycles (95°C/15s,54°C/30s,72°C/30s), 72°C/30s	[8]
	2R	GCIGARCARAAITCBGTRC		

Supplementary Table 5. Denitrifiers identified across paddy fields of China.

Denitrifiers	Survival condition	Ref	Denitrifiers	Survival condition	Ref
Caldilineaceae	anaerobic	9	*Phycisphaeraceae*	anaerobic	10
Cellulomonadaceae	facultatively anaerobic	11	*Ignavibacteriaceae*	anaerobic	12
Helicobacteraceae	microaerobic	13	*Gracilibacteria_fa*	anaerobic	14
Polyangiaceae	aerobic	15	*Veillonellaceae*	anaerobic	16
Bdellovibrionaceae	aerobic	17	*Ruminococcaceae*	anaerobic	18
Methylophilaceae	aerobic	19	*Peptococcaceae*	anaerobic	20
Alcaligenaceae	aerobic	21	*Lachnospiraceae*	anaerobic	22
Rhodobacteraceae	aerobic	23	*Clostridiaceae_1*	anaerobic	24
Xanthobacteraceae	aerobic	25	*JG30-KF-CM45_fa*	anaerobic	26
Rhizobiaceae	aerobic	25	*HSB_OF53-F07*	anaerobic	27
Cyclobacteriaceae	aerobic	28	*Chloroflexi_fa*	anaerobic	26
Gaiellaceae	aerobic	29	*Frankiaceae*	anaerobic	30
Euzebyaceae	aerobic	31	*Rhodospirillales_unclassified*	anaerobic	32
Mycobacteriaceae	aerobic	33	*Hyphomicrobiaceae*	anaerobic	34
Hyphomonadaceae	aerobic	35	*Gemmatimonadaceae*	facultatively aerobic	36
Sandaracinaceae	anaerobic	37	*Chloroflexaceae*	facultatively aerobic	38
Gallionellaceae	anaerobic	39	*Rhodocyclaceae*	facultatively anaerobic	40
Oxalobacteraceae	anaerobic	41	*Hydrogenophilaceae*	facultatively anaerobic	42
Comamonadaceae	anaerobic	43	*Caulobacteraceae*	facultatively anaerobic	11
Phyllobacteriaceae	anaerobic	44	*Bacillaceae*	facultatively anaerobic	45
Bradyrhizobiaceae	anaerobic	46	*Flavobacteriaceae*	facultatively anaerobic	47
Planctomycetaceae	anaerobic	48	*Cytophagaceae*	facultatively anaerobic	49

Supplementary Table 6. The associations of climate and soil with microbial attributes based on the structural equation modeling.

Parameters			Standardized regression weights	Regression weights	*P*
MAT	→	*nirK*	0.229	0.092	0.037
MAT	→	*nirS*	0.264	0.207	0.004
MAT	→	*pmoA*	0.253	0.079	0.048
MAP	→	*nirK*	-0.371	-1.879	< 0.001
MAP	→	*nirS*	-0.203	-1.998	0.031
MAP	→	*pmoA*	-0.322	-1.253	0.014
SOC	→	*nirK*	0.457	1.007	< 0.001
SOC	→	*nirS*	0.527	2.258	< 0.001
SOC	→	*pmoA*	0.343	0.581	< 0.001
pH	→	*pmoA*	0.227	1.637	0.023
NH_4^+	→	*nirS*	-0.281	-0.770	< 0.001
NH_4^+	→	NMDS1	0.235	0.193	0.028
pH	→	NMDS1	0.398	2.180	< 0.001

Supplementary Table 7. The description of genes involved in methane oxidation and denitrification.

Genes	Description	Genes	Description
pmo	particulate methane monooxygenase	*mmo*	soluble methane monooxygenase
edd	phosphogluconate dehydratase	*xpk*	xylulose-5-phosphate phosphoketolase
mxa	calcium-dependent methanol dehydrogenase	*xoxF*	lanthanide-dependent methanol dehydrogenase
fae	formaldehyde activating enzyme	*mtd*	methylene tetrahydromethanopterin
mch	methenyl tetrahydromethanopterin cyclohydrolase	*hxlA*	3-hexulose-6-phosphate synthase
hps	3-hexulose-6-phosphate synthase	*hxlB*	6-phospho-3-hexuloisomerase
pfk	6-phosphofructokinase	*aldo*	fructose-bisphosphate aldolase
ftr	formylmethanofuran-tetrahydromethanopterin	*mdo*	formaldehyde dismutase / methanol dehydrogenase
fdh	NAD-dependent formate dehydrogenase	*fdo*, *fdsD*, *fdw*	formate dehydrogenase major subunit
gly	serine hydroxymethyltransferase	*agxt*	serine-glyoxylate aminotransferase
hpr	hydroxypyruvate reductase	*gck*	glycerate kinase
eno	enolase	*ppc*	phosphoenolpyruvate carboxylase
madh	malate dehydrogenase	*mtk*	malate-CoA ligase
mcl	malyl-CoA lyase	*fum*	fumarate hydratase
sdh	succinate dehydrogenase	*suc*	succinyl-CoA synthetase
acnA	aconitate hydratase	*cs*	citrate synthase
pps	pyruvate, water dikinase	*por*	pyruvate ferredoxin oxidoreductase
pta	phosphate acetyltransferase	*ack*	acetate kinase
acs	acetyl-CoA synthetase	*gfa*	S-(hydroxymethyl)glutathione synthase
fadh	glutathione-independent formaldehyde dehydrogenase	*folD*	methylenetetrahydrofolate dehydrogenase
fchA	methenyltetrahydrofolate cyclohydrolase	*fhs*	formate--tetrahydrofolate ligase
tkt	transketolase	*tal*	transaldolase
rpe	ribulose-phosphate 3-epimerase	*gnt*	6-phosphogluconate dehydrogenase
ato	acetate CoA -transferase	*pct*	propionate CoA-transferase
nar/nap	periplasmic nitrate reductase	*nir*	copper-containing nitrite reductase
nor	nitric oxide reductase	*nosZ*	nitrous-oxide reductase

Supplementary References

1. Knief, C., Lipski, A. & Dunfield, P. F., Diversity and activity of methanotrophic bacteria in different upland soils. *Appl. Environ. Microbiol.* **69**, 6703-6714 (2003).
2. Costello, A. M. & Lidstrom, M. E., Molecular characterization of functional and phylogenetic genes from natural populations of methanotrophs in lake sediments. *Appl. Environ. Microbiol* **65**, 5066-5074 (1999).
3. Braker, G., Fesefeldt, A. & Witzel, K.-P., Development of PCR primer systems for amplification of nitrite reductase genes (nirK and nirS) to detect denitrifying bacteria in environmental samples. *Appl. Environ. Microbiol.* **64**, 3769-3775 (1998).
4. Throbäck, I. N., Enwall, K., Jarvis, Å. & Hallin, S., Reassessing pcr primers targeting nirS, nirK and nosZ genes for community surveys of denitrifying bacteria with DGGE. *FEMS. Microbiol. Ecol.* **49**, 401-417 (2004).
5. Kandeler, E., Deiglmayr, K., Tscherko, D., Bru, D. & Philippot, L., Abundance of narG, nirS, nirK, and nosZ genes of denitrifying bacteria during primary successions of a glacier foreland. *Appl. Environ. Microbiol.* **72**, 5957-5962 (2006).
6. Braker, G. & Tiedje, J. M., Nitric oxide reductase (norB) genes from pure cultures and environmental samples. *Appl. Environ. Microbiol.* **69**, 3476-3483 (2003).
7. Henry, S., Bru, D., Stres, B., Hallet, S. & Philippot, L., Quantitative detection of the nosZ gene, encoding nitrous oxide reductase, and comparison of the abundances of 16S rRNA, narG, nirK, and nosZ genes in soils. *Appl. Environ. Microbiol.* **72**, 5181-5189 (2006).
8. Jones, C. M., Graf, D. R., Bru, D., Philippot, L. & Hallin, S., The unaccounted yet abundant nitrous oxide-reducing microbial community: A potential nitrous oxide sink. *ISME. J.* **7**, 417-426 (2013).
9. Wu, L., Li, Z., Zhao, C., Liang, D. & Peng, Y. A novel partial-denitrification strategy for post-anammox to effectively remove nitrogen from landfill leachate. *Sci. Total. Environ.* **633**, 745-751 (2018).
10. Jasmin, C. et al. Diversity of sediment-associated planctomycetes in the arabian sea oxygen minimum zone. *J. Basic. Microbiol.* **57**, 1010-1017 (2017).
11. Garrity, G. M., Bell, J. A. & Lilburn, T. Caulobacteraceae. *Bergey's Manual of Systematics of Archaea and Bacteria* (Springer: Berlin, Heidelberg, 2015), pp. 1-25.
12. Zheng, Y. et al. Identifying microbial community evolution in membrane bioreactors coupled with anaerobic side-stream reactor, packing carriers and ultrasonication for sludge reduction by linear discriminant analysis. *Bioresource. Technol.* **291**, 121920 (2019).

13. Garrity, G. M., Bell, J. A. & Lilburn, T. Helicobacteraceae fam. Nov. *Bergey's Manual of Systematics of Archaea and Bacteria* (Springer: Berlin, Heidelberg, 2014), pp. 1-1 (2015).

14. Ji, J. et al. Effects of salinity build-up on the performance and microbial community of partial-denitrification granular sludge with high nitrite accumulation. *Chemosphere* **209**, 53-60 (2018).

15. Garcia, R. & Müller, R. The family polyangiaceae. *Bergey's Manual of Systematics of Archaea and Bacteria* (Springer: Berlin, Heidelberg, 2014), pp. 247-279.

16. Carlier, J.-P. et al. Anaeroglobus geminatus gen. Nov., sp. Nov., a novel member of the family veillonellaceae. *Int. J. Syst. Evol. Microbiol.* **52**, 983-986 (2002).

17. Sockett, R. E. & Lambert, C. Bdellovibrio as therapeutic agents: A predatory renaissance? *Nat. Rev. Microbiol.* **2**, 669-675 (2004).

18. Rainey, F. A. Family VIII. Ruminococcaceae fam. Nov. *Bergey's manual of systematic bacteriology* (Springer: Berlin, Heidelberg, 2009), pp.1016-1043.

19. Osaka, T. et al. Identification of acetate-or methanol-assimilating bacteria under nitrate-reducing conditions by stable-isotope probing. *Microb. Ecol.* **52**, 253-266 (2006).

20. Atashgahi, S. et al. A benzene-degrading nitrate-reducing microbial consortium displays aerobic and anaerobic benzene degradation pathways. *Sci. Rep.* **8**, 1-12 (2018).

21. Wang, H. et al. Breast tissue, oral and urinary microbiomes in breast cancer. *Oncotarget* **8**, 88122 (2017).

22. Rainey, F. A. Family V. Lachnospiraceae fam. Nov. *Bergey's manual of systematic bacteriology* (Springer: Berlin, Heidelberg, 2009), pp. 921-968 (2009).

23. Pujalte, M. J., Lucena, T., Ruvira, M. A., Arahal, D. R. & Macián, M. C. The family rhodobacteraceae. (Springer, 2014), pp. 439-512. (2014).

24. Heller, H. H. Certain genera of the clostridiaceae: Studies in pathogenic anaerobes. V. *J. Bacteriol.* **7**, 1-36 (1922).

25. Alves, L. M. C., De Souza, J. A. M., de Mello Varani, A. & de Macedo Lemos, E. The family rhizobiaceae. *The Prokaryotes*, 419-437 (Springer: Berlin, Heidelberg, 2014), pp. 419-437. (2014).

26. Nunoura, T. et al. Isolation and characterization of a thermophilic, obligately anaerobic and heterotrophic marine chloroflexi bacterium from a chloroflexi-dominated microbial community associated with a japanese shallow hydrothermal system, and proposal for thermomarinilinea lacunofontalis gen. Nov., sp.

Nov. *Microbes. Environ.* **28**, 228-235 (2013).

27. Lezcano, M. Á. et al. Biomarker profiling of microbial mats in the geothermal band of cerro caliente, deception island (antarctica): Life at the edge of heat and cold. *Astrobiology* **19**, 1490-1504 (2019).

28. Kumar, A. et al. Shivajiella indica gen. Nov., sp. Nov., a marine bacterium of the family "cyclobacteriaceae" with nitrate reducing activity. *Syst. Appl. Microbiol.* **35**, 320-325 (2012).

29. da Costa, L. A. M. S. 19 The family Gaiellaceae. *The. Prokaryotes-Actinobacteria* (Springer: Berlin, Heidelberg, 2014).

30. Woods, D. R. & Reid, S. J. Recent developments on the regulation and structure of glutamine synthetase enzymes from selected bacterial groups. *FEMS. Microbiol. Rev.* **11**, 273-283 (1993).

31. Xu, L. et al. Complete genome sequence of euzebya sp. Dy32-46, a marine actinobacteria isolated from the pacific ocean. *Mar. Genom.* **44**, 65-69 (2019).

32. López-López, A. et al. Extremely halophilic microbial communities in anaerobic sediments from a solar saltern. *Environ. Microbiol. Rep.* **2**, 258-271 (2010).

33. Magee, J. G. & Ward A. C. Family III. Mycobacteriaceae chester 1897, 63al. *Bergey's Manual of Systematic Bacteriology: Volume 5: The Actinobacteria* (Springer: Berlin, Heidelberg, 2012), pp. 312. (2012).

34. Oren, A. & Xu, X.-W. The family Hyphomicrobiaceae. *The Prokaryotes: Alphaproteobacteria and Betaproteobacteria* (Springer Berlin Heidelberg, Berlin, Heidelberg, 2014), pp. 247-281. (2014).

35. Lee, K., Lee, H. K., Choi, T.-H. & Cho, J.-C. Robiginitomaculum antarcticum gen. Nov., sp. Nov., a member of the family hyphomonadaceae, from antarctic seawater. *Int. J. Syst. Evol. Microbiol.* **57**, 2595-2599 (2007).

36. Hanada, S. & Sekiguchi, Y. in *The prokaryotes: Other major lineages of bacteria and the archaea,* E. Rosenberg, E. F. DeLong, S. Lory, E. Stackebrandt, F. Thompson, Eds. (Springer Berlin Heidelberg, Berlin, Heidelberg, 2014), pp. 677-681.

37. Mohr, K. I., Garcia, R. O., Gerth, K., Irschik, H. & Müller, R. Sandaracinus amylolyticus gen. Nov., sp. Nov., a starch-degrading soil myxobacterium, and description of sandaracinaceae fam. Nov. *Int. J. Syst. Evol. Microbiol.* **62**, 1191-1198 (2012).

38. Hanada, S. & Pierson, B. K. The family chloroflexaceae. *The prokaryotes* (Springer: Berlin, Heidelberg, 2006), pp. 815-842. (2006).

39. Bryce, C. et al. Microbial anaerobic Fe (II) oxidation–ecology, mechanisms and

environmental implications. *Environ. Microbiol.* **20**, 3462-3483 (2018).

40. Oren, A. in *The prokaryotes: Alphaproteobacteria and betaproteobacteria,* E. Rosenberg, E. F. DeLong, S. Lory, E. Stackebrandt, F. Thompson, Eds. (Springer Berlin Heidelberg, Berlin, Heidelberg, 2014), pp. 975-998.

41. Sydney, E. B. et al. Screening and bioprospecting of anaerobic consortia for biohydrogen and volatile fatty acid production in a vinasse based medium through dark fermentation. *Process. Biochem.* **67**, 1-7 (2018).

42. Luo, J.-F., Lin, W.-T. & Guo, Y. Functional genes based analysis of sulfur-oxidizing bacteria community in sulfide removing bioreactor. *Appl. Microbiol. Biot.* **90**, 769-778 (2011).

43. Willems, A. in *The prokaryotes: Alphaproteobacteria and betaproteobacteria,* E. Rosenberg, E. F. DeLong, S. Lory, E. Stackebrandt, F. Thompson, Eds. (Springer Berlin Heidelberg, Berlin, Heidelberg, 2014), pp. 777-851.

44. Moitinho-Silva, L. et al. Integrated metabolism in sponge–microbe symbiosis revealed by genome-centered metatranscriptomics. *ISME. J.* **11**, 1651-1666 (2017).

45. Márquez, M. et al. Aquisalibacillus elongatus gen. Nov., sp. Nov., a moderately halophilic bacterium of the family bacillaceae isolated from a saline lake. *Int. J. Syst. Evol. Microbiol.* **58**, 1922-1926 (2008).

46. de Souza, J. A. M., Carrareto Alves, L., de Mello Varani, A. & de Macedo Lemos, E. G. The family bradyrhizobiaceae. *The Prokaryotes* (Springer: Berlin, Heidelberg, 2014), pp. 135-154. (2014).

47. Bernardet, J.-F. & Nakagawa, Y. An introduction to the family Flavobacteriaceae. *The Prokaryotes: Volume 7: Proteobacteria: Delta, Epsilon Subclass* (Springer New York, NY, 2006), pp. 455-480. (2006).

48. Scheuner, C. et al. Complete genome sequence of planctomyces brasiliensis type strain (DSM 5305 T), phylogenomic analysis and reclassification of planctomycetes including the descriptions of gimesia gen. Nov., planctopirus gen. Nov. And rubinisphaera gen. Nov. And emended descriptions of the order planctomycetales and the family planctomycetaceae. *Stand. Genomic. Sci* **9**, 10 (2014).

49. Xuemei, M. J. M. W. L. & Zhang, L. Y. Z. W. 44 The Family Cytophagaceae. *The prokaryotes* (Springer: Berlin, Heidelberg, 2014), pp. 577-593. (2014).
