# Supplementary material for: Metabolic coupling between soil aerobic methanotrophs and denitrifiers in rice paddy fields
Source: Nat Commun. 2024 Apr 24;15:3471. doi: 10.1038/s41467-024-47827-y (PMC11043409; doi:10.1038/s41467-024-47827-y)
Supplement: Supplementary file 2 — Reporting Summary [file 41467_2024_47827_MOESM2_ESM.pdf]

nature portfolio

Corresponding author(s): Jiao Feng (fengjiao@mail.hzau.edu.cn)
Yu-Rong Liu (yrliu@mail.hzau.edu.cn)

Last updated by author(s): Apr 10, 2024

Reporting Summary

Nature Portfolio wishes to improve the reproducibility of the work that we publish. This form provides structure for consistency and transparency in reporting. For further information on Nature Portfolio policies, see our Editorial Policies and the Editorial Policy Checklist.

Statistics

For all statistical analyses, confirm that the following items are present in the figure legend, table legend, main text, or Methods section.

n/a	Confirmed	
☐	☒	The exact sample size (n) for each experimental group/condition, given as a discrete number and unit of measurement
☐	☒	A statement on whether measurements were taken from distinct samples or whether the same sample was measured repeatedly
☐	☒	The statistical test(s) used AND whether they are one- or two-sided<br>*Only common tests should be described solely by name; describe more complex techniques in the Methods section.*
☐	☒	A description of all covariates tested
☐	☒	A description of any assumptions or corrections, such as tests of normality and adjustment for multiple comparisons
☐	☒	A full description of the statistical parameters including central tendency (e.g. means) or other basic estimates (e.g. regression coefficient) AND variation (e.g. standard deviation) or associated estimates of uncertainty (e.g. confidence intervals)
☐	☒	For null hypothesis testing, the test statistic (e.g. F, t, r) with confidence intervals, effect sizes, degrees of freedom and P value noted<br>*Give P values as exact values whenever suitable.*
☒	☐	For Bayesian analysis, information on the choice of priors and Markov chain Monte Carlo settings
☒	☐	For hierarchical and complex designs, identification of the appropriate level for tests and full reporting of outcomes
☐	☒	Estimates of effect sizes (e.g. Cohen's d, Pearson's r), indicating how they were calculated

Our web collection on statistics for biologists contains articles on many of the points above.

Software and code

Policy information about availability of computer code

Data collection	Data for this manuscript were from field studies and lab analyses of 139 paddy field soils along a ~3300 km transect in the main rice-producing areas of China. Climatic variables were collected using ArcGIS V10.6 software. Soil properties, soil microbial activities associated with denitrification and methane oxidation activities in the field survey and microcosm experiments were measured as explained in the Method section of our manuscript. All code associated with our analyses in this study is available at: https://figshare.com/s/c1a8ad171646eae9b6c9.
Data analysis	The Fast Length Adjustment of Short Reads (FLASH) software (v1.2.11) was used to filter and assemble raw and paired-end sequences. A combination of QIME2 (v2020.2)and UPARSE (v7.1) were used for Bioinformatic analysis. The fastp software (https://github.com/OpenGene/fastp, v0.19.6) was used to filter sequences, and qualified reads were assembled into contigs using Megahit (https://github.com/voutcn/megahit, v1.2.9) for shotgun metagenomic analyses. DAS_tools (v 1.1.0) and CheckM (v1.1.6) were used to respectively dereplicate and evaluate all recovered bins to obtain non-redundant high-quality draft genomes. The coverage of metagenome-assembled genomes (MAGs) in each sample was estimated using CoverM (v0.6.1), and the relative abundance of each MAG is calculated using the Quant_bins module in MetaWRAP (v1.3). FastANI (v 1.0) was used to calculate the values of genome ANI. High-quality reads were aligned to the non-redundant gene catalogs to calculate gene abundance with 95% identity using SOAPaligner (v2.04). The predicted genes within MAGs were annotated using the HMM profile database for KEGG orthology with predefined score thresholds via KofamScan (v 1.3.0). The relationships between denitrification rates and methane-oxidizing activities were analyzed using "psych" package in R 4.0.2. The differences in soil denitrification rates and methane oxidation activities and genes in different treatments were conducted using One-way ANOVA followed by two-sided Tukey post hoc test by SPSS 21.0 (IBM, Chicago, IL). Structural equation modeling analyses were conducted using AMOS 21.0 (spsss Inc., Chicago, IL, USA). LEfSe analysis ($p < 0.05$, LDA score > 2) was used to identify bacterial biomarkers for two groups on the open website (http://huttenhower.sph.harvard.edu/galaxy). We used DESeq2 (v1.36.0) to identify OTUs significantly enriched in the heavy fractions of the 13C-labeled treatments compared to the heavy fractions in the corresponding 12C controls. All metabolite identification and isotopic enrichment were determined by MassHunter Workstation Software (v B.08.00, Agilent) and ChemStation Software (v E.02.02.1431, Agilent) using the

default parameters and assisting manual inspection.

For manuscripts utilizing custom algorithms or software that are central to the research but not yet described in published literature, software must be made available to editors and reviewers. We strongly encourage code deposition in a community repository (e.g. GitHub). See the Nature Portfolio guidelines for submitting code & software for further information.

Data

Policy information about availability of data

All manuscripts must include a data availability statement. This statement should provide the following information, where applicable:

- Accession codes, unique identifiers, or web links for publicly available datasets
- A description of any restrictions on data availability
- For clinical datasets or third party data, please ensure that the statement adheres to our policy

The gene amplicon sequences and metagenomic sequences generated in this study have been deposited to the NCBI SRA database under the BioProject IDs of PRJNA1096118, PRJNA1097355 and PRJNA1096656. The databases used in this study include Worldclim database (https://www.worldclim.org), SILVA 128 (https://www.arb-silva.de/documentation/release-128/), NcycFunGen database (http://mem.rcees.ac.cn:8088/), Genome Taxonomy Database (http://gtdb.ecogenomic.org/) and KEGG database (https://ww.genome.ad.jp/kegg/).
Additional figures and tables can be found in the Supporting Information. Source data are provided with this paper.

Research involving human participants, their data, or biological material

Policy information about studies with human participants or human data. See also policy information about sex, gender (identity/presentation), and sexual orientation and race, ethnicity and racism.

Reporting on sex and gender	N/A
Reporting on race, ethnicity, or other socially relevant groupings	N/A
Population characteristics	N/A
Recruitment	N/A
Ethics oversight	N/A

Note that full information on the approval of the study protocol must also be provided in the manuscript.

Field-specific reporting

Please select the one below that is the best fit for your research. If you are not sure, read the appropriate sections before making your selection.

☐ Life sciences ☐ Behavioural & social sciences ☒ Ecological, evolutionary & environmental sciences

For a reference copy of the document with all sections, see nature.com/documents/nr-reporting-summary-flat.pdf

Ecological, evolutionary & environmental sciences study design

All studies must disclose on these points even when the disclosure is negative.

Study description	This study is based on a combination of field survey (139 paddy field soils along a ~3300 km transect) and lab analyses to investigate the coupling between soil aerobic methane oxidation and denitrification in the main rice-producing areas of China. The field survey provide evidence on the potential linkages between the two essential processes across large scale, and microcosm experiments of methane and aerobic methanotrophs addition further confirmed the linkages observed in the fields. Additionally, 13CH4-DNA-stable isotope probing (SIP)-metagenomics and 13C-metabolomics analyses identified key microbial taxa and metabolic pathways driving the coupling between soil aerobic CH4 oxidation and denitrification. The DNA-SIP was conducted with three replicates using 13C-labeled or unlabeled CH4 as C sources. The 13C-metabolomics were conducted with three treatments (three replicates), including no addition of CH4 as control, the addition of 10% 13CH4, and the simultaneous addition of 10% 13CH4 and 100 mg kg−1 NO3--N. Our findings provide the attempt to investigate whether and how aerobic CH4 oxidation couples with denitrification in hypoxic paddy fields.

Research sample	In order to obtain soils that are representative of soil-rice systems on a national scale, we collected surface soils (top 15 cm) from the 139 paddy field soils from 12 provinces in China. These provinces account for over 90% of the total rice production areas in the temperate, subtropical and tropical regions of China. Soil properties, microbial community analyses and microbial activities (both activities and gene abundances) associated with aerobic methane oxidation and denitrification were measured in all 139 samples. For microcosm experiments (including methane and methanotroph addition), three typical soils collected from temperate, subtropical and tropical regions were used to investigate the effects of aerobic methane oxidation on denitrification. We did not analyze all soils for microcosm experiments due to that many of the analyses included (e.g., SIP-metagenomics and 13C-metabolomics) are time-consuming and highly costly. We thus focused on typical soils to investigate key microbial taxa and metabolic pathways driving the coupling between soil aerobic CH_4 oxidation and denitrification. These typical soil samples were selected to represent the main climatic regions across a broad range of environmental gradients of the main rice-producing areas of China.
Sampling strategy	When determining our sample sizes, we carefully considered various factors to ensure the robustness and representativeness of our data. Guided by previous research emphasizing the need for adequate sample sizes for reliable results, we carefully considered the complexity of the ecosystems by setting samples to extensive coverage different environmental gradients, including different climatic regions. To achieve this, we collected soil samples across a broad environmental gradient, covering over 90% of the total rice production areas in the temperate, subtropical, and tropical regions of China. At each site, we established five 1 m × 1 m sub-plots positioned at each corner and center of a 50 m × 50 m area. Five soil cores (top 15 cm) were collected and then mixed into a composite soil sample to consider the heterogeneity. This approach enabled us to capture the variability in soil properties across the study area. Additionally, conducting soil sampling under waterlogged conditions allowed us to obtain representative samples reflective of the typical conditions found in paddy fields. We ensured that our sample sizes were sufficient to detect meaningful effects with adequate statistical power, reducing the risk of false negatives. This sampling strategy ensured that our data accurately represented the prevailing environmental conditions and microbial communities in these ecosystems. Detailed information on the sampling strategy was shown in Supplementary Fig. 6 and the Method section.
Data collection	In the field survey, Kang-Hua Chen measured soil denitrification rates and methane-oxidizing activities as the amount of reaction products per g soil in per unit time using isotope ratio mass spectrometry (MAT 253 plus, Thermo, USA) and Agilent GC7890A gas chromatograph, respectively. Kang-Hua Chen measured all soil properties. Specifically, soil pH was determined on a 1: 2.5 soil/water extract using a pH electrode. Soil organic carbon was determined by the potassium dichromate oxidation titration method. Ammonium and nitrate were measured by the KCl extraction method. Microbial biomass carbon was measured using the fumigation-extraction method. For bacterial community composition analyses, Kang-Hua Chen extracted soil DNA, and soil DNA was characterized by Illumina Miseq sequencing of the V3-V4 region of the 16S rRNA gene amplification at Majorbio in Shanghai, China.<br><br>In CH_4 and methanotroph addition experiments, Kang-Hua Chen measured the gases using Agilent GC7890A gas chromatograph, extracted soil total RNA using the RNeasy PowerSoil Total RNA Kit, converted RNA to complementary DNA using a PrimeScriptTM RT reagent Kit with gDNA Eraser (TaKaRa), and used real-time quantitative PCR to measure key functional genes responsible for aerobic methane oxidation (pmoA gene) and denitrification (nirK and nirS genes). The pmoA, nirK and nirS gene amplicons were sequenced at Majorbio in Shanghai, China. Following the sequence, Kang-Hua Chen performed the subsequent data analysis.<br><br>Kang-Hua Chen conducted the $^{13}CH_4$-DNA-SIP experiment by a CsCl gradient ultracentrifugation method combined with Illumina Miseq sequencing. Heavy DNA fractions from the $^{13}CH_4$-incubated samples were further used for shotgun sequencing at Majorbio in Shanghai, China. Following the sequence, Kang-Hua Chen performed the subsequent data analysis to investigate the metabolic pathways associated with the coupling between aerobic methane oxidation and denitrification.<br><br>In 13C-metabolomics experiments, both LC-MS/MS and GC-MS experiments were performed at ProfLeader in Shanghai, China. Kang-Hua Chen performed the subsequent data analysis to identify the intermediates derived from the oxidation of $^{13}CH_4$ that support denitrification.
Timing and spatial scale	Sample collection of soils of 139 paddy soils along a ~3300 km transect of the main rice-producing areas of China predominantly took place in 2018 (from November 2017 to March 2019). The methane addition experiment was sampled on days 0, 1, 2, 4, 6, 10, 12 and 15. The methanotroph addition experiment was sampled on days 0, 1, 2, 3 and 5. The DNA-SIP experiment was sampled on days 0, 15 and 40.
Data exclusions	No data were excluded in the analyses.
Reproducibility	Within the manuscript, we clearly state all the steps taken to ensure the reproducibility of the study. We include descriptions of standard sampling and analytical protocols and the identification of all code packages used. All microcosm experiments were conducted with three biological replicates. Each experiment was performed twice per condition of the experiment, and all attempts to repeat the experiment were successful.
Randomization	N/A
Blinding	N/A

Did the study involve field work? ☒ Yes ☐ No

Field work, collection and transport

Field conditions	Field sampling was conducting a wide range of environmental conditions, and the mean annual precipitation and temperature. ranged from 493 to 1861 mm and 2.9 °C to 25.0 °C, respectively. More details can be found in the Methods section of the manuscript.

Location	Detailed information on geographical coordinates of sampling locations included in this study were presented in Supplementary Fig. 1 and Methods. The geographical information of three typical soils for microcosm experiments were shown in Supplementary Table 1. The sample sites distribute in the main rice-producing areas of China, with latitude ranging from 19.99 °N to 47.24 °N and longitude ranging from 102.74 °E to 130.59 °E.
Access & import/export	All soil samples collected by our co-authors from the main rice-producing areas completely comply with local law, and no permission is needed for sample collection.
Disturbance	This study did not cause any environmental disturbance.

Reporting for specific materials, systems and methods

We require information from authors about some types of materials, experimental systems and methods used in many studies. Here, indicate whether each material, system or method listed is relevant to your study. If you are not sure if a list item applies to your research, read the appropriate section before selecting a response.

Materials & experimental systems

n/a	Involved in the study
☒	☐ Antibodies
☒	☐ Eukaryotic cell lines
☒	☐ Palaeontology and archaeology
☒	☐ Animals and other organisms
☒	☐ Clinical data
☒	☐ Dual use research of concern
☒	☐ Plants

Methods

n/a	Involved in the study
☒	☐ ChIP-seq
☒	☐ Flow cytometry
☒	☐ MRI-based neuroimaging

Plants

Seed stocks	N/A
Novel plant genotypes	N/A
Authentication	N/A
